# Does Epstein-Barr virus infection have an influence on the development of laryngeal carcinoma? Detection of EBV by *Real-Time Polymerase Chain Reaction* in tumour tissues of patients with laryngeal carcinoma

**DOI:** 10.5935/1808-8694.20130075

**Published:** 2015-10-08

**Authors:** Tuba Muderris, Seyyal Rota, Togay Muderris, Erdogan İnal, Isıl Fidan

**Affiliations:** a Microbiologist.; b Professor (Head, Department of Microbiology, Gazi University Faculty of Medicine, Ankara, Turkey).; c Specialist (Otorhinolaryngologist).; d Professor (Department of Otolaryngology, Gazi University Faculty of Medicine, Ankara, Turkey).; e Associate Professor (Department of Microbiology, Gazi University Faculty of Medicine, Ankara, Turkey).

**Keywords:** Epstein-Barr virus infections, laryngeal neoplasms, polymerase chain reaction

## Abstract

Epstein-Barr virus (EBV) is a well-known carcinogenic virus, and the association of EBV with some tumours suggests that there may also be an association between laryngeal carcinoma and EBV.

**Objective:**

The aim of this study is to determine the role of EBV in the aetiology of laryngeal carcinoma.

**Method:**

Prospective investigation the EBV with real time polymerase chain reaction in tumour tissues of 25 patients with laryngeal carcinoma and 17 patients with benign laryngeal lesions, and investigation of the relationship between the presence of viral DNA and patients’ smoking habits, alcohol consumption, localization and differentiation of the tumour.

**Results:**

There was no significant difference between the control group and patient group in terms of EBV polymerase chain reaction positivity (*p* > 0.05). Also we couldn't find a statistically significant relationship between EBV positivity and differentiation of the tumour, localization of the tumour, smoking and alcohol consumption habits (*p* > 0.05).

**Conclusion:**

Our results suggest that, although EBV is present in some of the squamous cell laryngeal carcinomas, its presence has no effect on the pathogenesis of laryngeal carcinomas.

## INTRODUCTION

Head and neck malignancies accounts for 4% of all types of cancers, and laryngeal carcinoma accounts for 25% to 40% of head and neck malignancies^1^. The role of many factors, especially tobacco use and alcohol consumption, has been clearly shown in the development of laryngeal carcinoma. Also it is known that certain viruses have oncogenic potentials, and the relationship between laryngeal carcinoma and viruses has been a popular subject of research for many years^2^, ^3^, ^4^.

Epstein-Barr virus (EBV) is present in all populations, infecting more than 95% of human beings within the first decade of life^5^. The host range of the *Lymphocryptovirus genus*, which also includes EBV, is generally restricted to primate B lymphocytes, which are also the site of latent virus infection *in vivo*. Infection of primate B-lymphocytes with lymphocryptoviruses typically results in a latent infection characterized by persistence of the viral genome with expression of latent gene products that contribute to the transformation process and cell proliferation^6^. The close relationship between EBV infection and nasopharyngeal carcinoma has been widely accepted^7^. Carcinomas that share the histological features of undifferentiated nasopharyngeal carcinomas have been identified in other sites of body, including the thymus, larynx, tonsils, salivary glands, lungs, skin, uterine cervix, bladder, and stomach^2^, ^7^, ^8^, ^9^, ^10^, ^11^, ^12^, ^13^, ^14^, ^15^, ^16^, ^17^, ^18^, ^19^, ^20^, ^21^, ^22^, ^23^. Recently, studies have reported polymerase chain reaction detection of EBV in a significant percentage of breast and hepatocellular carcinomas^5^, ^7^, ^8^, ^23^. Also some studies showed a possible role of EBV in the development of squamous cell laryngeal carcinoma^8^.

### Objective

We investigated the DNA of the EBV with a sensitive and specific molecular method, real time polymerase chain reaction (RT-PCR), in tumour tissues of patients with laryngeal carcinoma to determine the role of EBV in the aetiology of laryngeal carcinoma. We also analyzed the relationship between the presence of viral DNA and patients’ smoking habits, alcohol consumption, localization (glottic, supraglottic or subglottic) and differentiation of the tumour (well, moderate, poor).

## METHOD

Samples taken from fresh tumour tissues of randomly selected 25 patients that attended to the Department of Otolaryngology-Head and Neck Surgery between November 2007 and November 2008 with complaints of hoarseness, dyspnea, cough, sore throat and diagnosed as laryngeal carcinoma based on pathology results following laryngectomy or biopsy were included to the study.

Control group was composed of fresh tissue samples taken from patients that operated for benign laryngeal lesions like laryngeal polyps, nodules, cysts or granulomas. Also, biopsies taken from patients with diagnosis of laryngeal cancer that subsequently revealed to be a benign lesion after pathologic examination were included to the control group. A total of 17 samples obtained from patients with benign lesions consisted the control group. Biopsies taken from premalignant lesions like leukoplakia or dysplasia were not included to the study. The study was done with the approval of ethics committee of the institution. (approval nº 09-230). Every patient was informed about the study preoperatively and a signed consent was taken.

All samples were taken in operation room from fresh tissue biopsies just before formalin fixation of the tissue, in a sterile manner to avoid contamination risk.

Patients underwent thorough head and neck examination including indirect laryngoscopic evaluation and they were assessed in terms of localization of the tumour, smoking and alcohol consumption habits, duration of smoking and alcohol consumption and histopathological type of the tumour. Data for smoking and alcohol consumption habits were collected preoperatively with the help of a specific questionnaire that included questions about the duration and amount of consumption. Necessary imaging studies were done in patients suspected to have cancer before direct laryngoscopic biopsy.

Presence of EBV DNA in tissue samples was investigated by quantitative PCR using RT-PCR technique.

### Obtaining DNA

EBV DNAs were obtained from the samples using QIA amp DNA minikit (Qiagen, Germany) in accordance with the user manual of the kit.

### Multiplication of DNA

DNAs obtained from tissue samples were multiplied with Rotor-Gene 6000 (Corbett research, Australia) device using Qiagen Artus EBV RT PCR kit (Catalog Number 4501263) (Lot Number 130162115) (Sensitivity 3.8 copy/µl). In every study, one negative control was used to avoid contamination risk.

### Evaluation of Data

Data analyses were performed using Software version Rotor-Gene 1.7.75. EBV quantitation kit included 2 fluorescent dyes, JOE and FAM. While JOE ensures visible internal control, FAM indicates EBV DNA positivity. JOE is checked in yellow channel at a wavelength of 530-555 and FAM is checked in green channel at a wavelength of 470-510.

Results were interpreted as:

1. If FAM channel is positive: EBV DNA is positive in the sample. If positivity is very high, JOE channel may be negative;
2. If FAM channel is negative and JOE channel is positive, EBV DNA is negative in the sample. If JOE channel is negative too, the reaction was thought to be inhibited by an inhibitor and thus analysis was repeated.

### Statistical analysis

The Chi-square test was used to compare the EBV PCR positivity of the study and control groups and to determine the association of EBV PCR positivity and patients’ smoking habits, alcohol consumption, localization of the tumour (glottic, supraglottic, subglottic) and differentiation of tumour tissue (well, moderately, poorly). All statistical calculations were performed using commercially available software (SPSS version 15.0 for Windows; SPSS Inc, Chicago, Illinois) and *p* < 0.05 was considered to be significant.

## RESULTS

Samples taken from 25 male patients (aged between 42 to 67 years, with a mean of 54.6) that attended to Otorhinolaryngology clinic and diagnosed as laryngeal carcinoma after direct laryngoscopic biopsy were included to the study. Samples were taken during partial or total laryngectomy in 13 patients and during laryngoscopic biopsy in 12 patients. Control group was composed of tissue samples taken from 17 patients (mean age 48.8, 13 males (76.5%) and four females (23.5%)) that had attended to clinic with hoarseness and found to have a benign laryngeal lesion (eg. laryngeal polyp, granuloma, cyst) after laryngoscopic examination and biopsy results. After head and neck examinations and direct laryngoscopy, patients that had laryngeal carcinoma were divided into 3 groups as glottic, supraglottic and subglottic based on the localization of the tumour. The tumour was glottic in 64% (16/25) of the patients, while supraglottic laryngeal carcinoma was detected in 36% (9/25) of them. No subglottic lesion was detected in our study group.

Tissue samples taken from the patients were studied by quantitative PCR using RT-PCR technique for detection of EBV DNA. Internal control was found to be positive in all of the patients.

EBV PCR positivity was found in 40% (10/25) of the laryngeal carcinoma patients. EBV DNA was £10^3^ copy/ml in three patients, 10^3^-10^5^ copy/ml in six patients and 10^5^ copy/ml in one patient. It was positive in 66.7% (6/9) of the supraglottic tumours and in 25% (4/16) of the glottic tumours. In the control group, 52.9% (9/17) of the patients were EBV PCR negative while the remaining 47.1% (8/17) were EBV PCR positive. In five of these patients, EBV DNA was £10^3^ copy/ml, and in the remaining three, EBV DNA was 10^3^-10^5^ copy/ml. There was no significant difference between the control group and patient group in terms of EBV PCR positivity, and no direct correlation was found between EBV and the pathogenesis of laryngeal squamous cell carcinoma (SCC) (*p* > 0.05) (Table 1). Also, there was no significant relationship between EBV DNA positivity and localization of the tumour (*p* > 0.05).

**Table 1:** EBV positivity rates of the patients and control group and the association between EBV positivity, localization and differentiation of the tumour.

		EBV negative	EBV positive	*p*
Cancer patients (N: 25)		15 (60%)	10 (40%)	0.892
Control group (N: 17)		9 (52.9%)	8 (47.1%)	

Localization of the tumour	Glottic	12 (75%)	4 (25%)	0.087
	Supraglottic	3 (33.3%)	9 (66.7%)	
	Poorly differentiated	2 (40%)	3 (60%)	
Differentiation of the tumour	Moderately differentiated	5 (62.5%)	3 (37.5%)	0.328
	Well differentiated	8 (72.7%)	3 (27.3%)	
	Basaloid type	0 (0%)	1 (100%)	

Pathologic investigation of the samples taken from the patients revealed that, 44% (11/25) of the patients had well differentiated SCC, 32% (8/25) had moderately differentiated SCC, 20% (5/25) had poorly differentiated SCC and 4% (1/25) had basaloid type SCC. EBV DNA positivity was found in 27.3% (3/11) of the patients that had well differentiated SCC, in 37.5% (3/8) of the patients that had moderately differentiated SCC, in 60% (3/5) of the patients that had poorly differentiated SCC and it was also positive in the only patient that had basaloid type SCC (Table 1). We could not find a statistically significant relationship between EBV positivity and differentiation of the tumour (*p* > 0.05).

Patients that have cancer and controls were interviewed for determination of smoking and alcohol consumption habits. 96% (24/25) of the patients in the laryngeal carcinoma group were smokers while there was only one patient who was a non-smoker. Smoking period ranged from 15 to 43 years (mean 30 ± 4.5 years) with 20 to 40 cigarettes daily (mean 24.5 ± 8.25 cigarettes). Sixty percent (15/25) of the patients were drinking alcohol on regular basis (at least twice a week) for a duration of 10 to 30 years (mean duration of alcohol consumption was 22.5 years). In the control group, 76.5% (13/17) of the patients were smokers. Smoking period ranged from 14 to 39 years (mean 27.3 ± 3.8 years) with 20 to 40 cigarettes daily (mean 23.5 ± 7.5 cigarettes). Of these patients, 17.6% (3/17) were found to consume alcohol regularly and the mean duration of alcohol consumption was 28.3 years (20 to 35 years). EBV DNA positivity was 37.5% (9/24) in tissue samples of smoking cancer patients. In control group, EBV DNA positivity was found to be 46.2% (6/13) in smokers. In cancer group, samples taken from the patients that consume alcohol revealed an EBV DNA positivity of 40% (6/15). EBV DNA was not found in samples taken from control patients that consume alcohol. There was no statistically significant association between EBV DNA positivity and smoking and alcohol consumption habits (*p* > 0.05) (Table 2).

**Table 2:** Tobacco and alcohol consumption characteristics and EBV positivity rates of the patients.

	Cigarette	EBV negative	EBV positive	*p*
Control group	(-)	2 (50%)	2 (50%)	1.000
(N: 17)	(+)	7 (53.8%)	6 (46.2%)	

Cancer patients	(-)	0 (0%)	1 (100%)	0.400
(N: 25)	(+)	15 (62.5%)	9 (37.5%)	

	Alcohol	EBV negative	EBV positive	*p*

Control group	(-)	6 (42.9%)	8 (57.1%)	0.206
(N: 17)	(+)	3 (100%)	0 (0%)	

Cancer patients	(-)	6 (60%)	4 (40%)	1.000
(N: 25)	(+)	9 (60%)	6 (40%)	

## DISCUSSION

EBV is present in all populations, infecting more than 95% of human beings within the first decades of life^7^. In developing countries, certain cultural practices often lead to EBV exposure in early childhood, and primary EBV infection in young children is typically associated with an unremarkable acute syndrome. In more developed countries, however, infection is often delayed, and acute primary EBV infection occurring in adolescence or adulthood can result in a self-limiting lymphoproliferative disorder known as infectious mononucleosis (IM)^6^.

Data from several studies suggests that EBV is involved in the development or progression of squamous cell carcinoma of the nasopharynx, oral cavity, larynx and esophagus, as well as in gastric epithelioma and Hodgkin's lymphoma^8^, ^9^, ^10^, ^11^, ^12^, ^13^, ^14^, ^15^, ^16^, ^23^, ^24^, ^25^. In addition, it has also been implicated in the etiologies of the African type Burkitt's lymphoma, thymic carcinoma and Sjögren's syndrome^17^, ^19^.

The strongest environmental factor in the pathogenesis of laryngeal carcinoma is smoking. Gastroesophageal reflux, radiation, consuming fruits and vegetables which are rich in carotenoids and exposure to wood dust, heavy metals and coal dust are among suspected etiological factors^26^. Additionally, it has been thought that viral factors could play a role in the etiology of laryngeal carcinoma too. Some studies, with different results, have been conducted on the role of viral factors, mainly investigating the effects of Human Papillomavirus (HPV) and EBV in the etiology of laryngeal carcinoma^27^. Although various HPV types have been shown in samples taken from laryngeal carcinoma patients in some studies, HPV has not been considered to have a strong carcinogenic effect in development of laryngeal carcinomas because these observations were exceptional^28^, ^29^. Recently, there have been some reports presenting the association of EBV with laryngeal carcinoma^8^, and a number of reports refuting these data^19^, ^30^.

Gök et al.^20^ investigated the presence of EBV DNA in formalin-fixed, paraffin-embedded tissue samples from 22 patients with squamous cell carcinoma of the larynx and from 17 patients with vocal cord nodules by PCR. Polymerase chain reaction showed EBV DNA in 11 patients (50%) with laryngeal carcinoma and in seven patients (41.2%) with vocal cord nodules. They could not find any significant difference between groups in terms of EBV DNA positivity and duration of smoking, the number of cigarettes consumed daily, localization of the disease and tumour stage, which are consistent with our results.

Goldenberg et al.^21^ also could not find any significant relationship between EBV and tumour development in the study they performed on three hundred patients with head and neck cancer, including larynx, hypopharynx, oropharynx and oral cavity tumours^21^. They also could not find any correlation between EBV positivity and tobacco exposure, alcohol consumption or tumour grade. They found low quantities of EBV detected in a minority of head and neck cancers and they connected this to the presence of EBV genome in rare lymphoid or epithelial cells adjacent to the primary head and neck cancer.

In the study of de Oliveira et al.^2^, EBV was studied with molecular biological techniques in parafinnized tumour tissues taken from 110 patients having squamous cell laryngeal carcinoma, and EBV was detected in none of the patients. Similarly, Atula et al.^19^ suggested that EBV was not associated with laryngeal carcinoma after they analyzed EBV DNA in 79 frozen biopsy samples of head and neck cancer patients with Southern blot hybridization and PCR.

In their study, Vlachtsis et al.^22^ demonstrated EBV DNA positivity in 39 (43.3%) of 90 laryngeal SCC patients while both HPV and EBV positivity was found in 19 (21.1%) of them. It is impossible to determine the effect of EBV on laryngeal carcinoma regarding to their results because they did not have a control group, but their EBV DNA positivity rate in laryngeal SCC was similar to what we found.

Kiaris et al.^8^ have also studied the incidence of EBV in SCC of larynx. They analyzed EBV DNA presence by sensitive PCR and used RFLP (restriction fragment length polymorphism) for further confirmation of the specificity of the PCR-amplification reaction. EBV DNA was positive in 9 of the 27 tumour tissues while only four (15%) specimens from adjacent normal tissue exhibited evidence of EBV infection. Three samples were EBV positive for both normal and tumour tissue. Researchers have found a relatively high incidence of EBV in the tumour tissue (33%) of patients with laryngeal cancer, as compared to the low incidence (15%) of the virus genome detected in the adjacent normal tissue, which indicates a probable role of EBV in the development of the disease. However, they found no association with EBV positivity and stage of the disease and histological differentiation.

In our study, we could not find a significant relationship between the control group and patient group in terms of EBV PCR positivity and viral load of EBV. Similarly there was no direct association between EBV and pathogenesis of laryngeal squamous cell carcinoma. Most of the studies that performed previously support our results, but there are few studies that demonstrate an association between EBV and laryngeal SCC. We believe that such contradictory results are due to the small sample size and variety in sensitivity and specificity of the methods used for determination of EBV.

We also could not find a significant association between EBV positivity and localization and differentiation of the tumour (*p* > 0.05). Furthermore, since smoking and alcohol consumption are well-established risk factors for development of laryngeal carcinoma, we investigated the association between EBV positivity and these factors, and we could not demonstrate any relationship between smoking and alcohol consumption habits and both EBV positivity and viral load (*p* > 0.05). These results suggest that EBV does not play a synergistic role in development of laryngeal carcinomas with irritative factors like smoke and alcohol.

The main advantage of our study was the usage of fresh tissue samples for the determination of EBV DNA. Most of the previous studies investigating presence of EBV were performed on samples taken from formalin fixed, paraffin embedded tissues and formalin is a known inhibitor for PCR^31^. Hence, some false negative results could have been obtained in some of the previous studies.

In this study, surgical specimens of patients with benign laryngeal lesions were accepted as controls since taking tissue samples from healthy volunteers was not reasonable. Interestingly, EBV DNA was found to be positive in 47.1% of patients in this group, and this ratio was higher than the study group. So, it can be suggested that EBV is a very common virus that could stay latent in mucosal cells of the upper airway in considerable proportion of the population.

## CONCLUSION

Recently established association of EBV especially with the non-differentiated nasopharyngeal carcinoma has let to the consideration that there may also be an association between laryngeal carcinoma and EBV. But our results, in concordance with the results of the majority of the previous studies, suggest that, EBV is a very common virus that can be found in the mucosal cells of the upper airway in considerable proportion of the population, and although EBV is present in cancer tissues of some of the squamous cell laryngeal carcinomas, its presence has no effect on the pathogenesis of laryngeal carcinomas. Further multicentric studies with large sample sizes have to be performed to demonstrate the relationship between EBV and squamous cell laryngeal carcinoma clearly. By this way, with any possible association that could be found, positive steps can be taken in terms of prevention and management of laryngeal carcinomas.
